# Intraoperative Nomograms, Based on One-Step Nucleic Acid Amplification, for Prediction of Non-sentinel Node Metastasis and Four or More Axillary Node Metastases in Breast Cancer Patients with Sentinel Node Metastasis

**DOI:** 10.1245/s10434-018-6633-0

**Published:** 2018-07-05

**Authors:** Kenzo Shimazu, Nobuaki Sato, Akiko Ogiya, Yoshiaki Sota, Daisuke Yotsumoto, Takashi Ishikawa, Seigo Nakamura, Takayuki Kinoshita, Hitoshi Tsuda, Yasuyo Ohi, Futoshi Akiyama, Shinzaburo Noguchi

**Affiliations:** 1 Department of Breast and Endocrine Surgery, Osaka University Graduate School of Medicine, Osaka, Japan; 2 Department of Breast Oncology, Niigata Cancer Center Hospital, Niigata, Japan; 3 Department of Surgical Oncology, Breast Oncology Center, Cancer Institute Hospital of the Japanese Foundation for Cancer Research, Tokyo, Japan; 4 Department of Breast Surgical Oncology, Hakuaikai Sagara Hospital, Kagoshima, Japan; 5 Department of Breast Oncology, Tokyo Medical University Hospital, Tokyo, Japan; 6 Division of Breast Surgical Oncology, Department of Surgery, Showa University School of Medicine, Tokyo, Japan; 7 Breast Surgery Division, National Cancer Center Hospital, Tokyo, Japan; 8 Department of Basic Pathology, National Defense Medical College, Tokorozawa, Japan; 9 Department of Pathology, Hakuaikai Sagara Hospital, Kagoshima, Japan; 10 Department of Pathology, Cancer Institute Hospital, Japanese Foundation for Cancer Research, Tokyo, Japan

## Abstract

**Background:**

One-step nucleic acid amplification (OSNA) for cytokeratin 19 messenger RNA is an intraoperative diagnostic procedure for the detection of lymph node metastasis.

**Objective:**

This study aimed to construct intraoperative nomograms using OSNA for the prediction of non-sentinel lymph node (NSLN) metastasis and four or more axillary lymph node (ALN) metastases.

**Methods:**

Of the 4736 breast cancer patients (T1-3, N0) who underwent sentinel lymph node (SLN) biopsy and had SLNs examined intraoperatively with OSNA, 623 with SLN metastasis treated with completion ALN dissection (cALND) were retrospectively analyzed, and were randomly divided into training (*n* = 312) and validation (*n* = 311) sets.

**Results:**

Of the clinicopathological parameters available preoperatively and intraoperatively, the multivariate analysis of the training set revealed that clinical tumor size and total tumor load (TTL) determined by OSNA were significantly associated with NSLN metastasis, and that clinical tumor size, number of macrometastatic SLNs, and TTL were significantly associated with four or more ALN metastases. Nomograms for NSLN metastasis and four or more ALN metastases were constructed using these parameters, and their area under the receiver operating characteristic curve (AUC) of the validation set were both 0.70, with a diagnostic accuracy similar to that of previously reported postoperative nomograms.

**Conclusions:**

We constructed intraoperative nomograms using OSNA for the prediction of NSLN metastasis and four or more ALN metastases. These nomograms are as accurate as the conventional postoperative nomograms and might be helpful for decision making regarding the indication for cALND or the choice of adjuvant chemotherapeutic regimens and radiation field.

**Electronic supplementary material:**

The online version of this article (10.1245/s10434-018-6633-0) contains supplementary material, which is available to authorized users.

Axillary lymph node (ALN) status is still the most important prognostic factor for patients with breast cancer, and sentinel lymph node biopsy (SLNB) is currently widely accepted as a standard surgical procedure for the determination of ALN status of clinically node-negative breast cancer patients.^1^–^5^ In case of negative sentinel lymph node (SLN), completion ALN dissection (cALND) can be avoided; however, in case of positive SLN, cALND is performed because of a high probability of non-SLN (NSLN) metastasis, whereas a certain proportion of patients with positive SLN are forgoing cALND if they meet the inclusion criteria for the American College of Surgeons Oncology Group (ACOSOG) Z0011 study.^6^,^7^ It has been reported that 40–70% of SLN-positive patients actually have NSLN metastasis,^8^ indicating that approximately half of SLN-positive patients undergo unnecessary cALND. In the current era of precision medicine, the risk of NSLN metastasis should be estimated accurately, and the indication for cALND should be personalized according to the risk of NSLN metastasis. For these reasons, various prediction models for NSLN metastasis and four or more ALN metastases have been developed for SLN-positive breast cancer patients.^9^–^18^

Such prediction models mainly consist of pathological parameters of tumor (size, lymphovascular invasion [LVI]) and SLN (size of metastatic deposit, number of involved SLNs).^9^,^15^ However, these models have certain limitations in that they can only be used postoperatively because they incorporate the pathological parameters that can only be obtained after pathological examination of the resected specimens. Therefore, a disadvantage of these models is that a patient who postoperatively turns out to have a high probability of NSLN metastasis on the basis of a prediction model needs to undergo a second surgery, which can be associated with patient distress, additional cost, and delay of adjuvant therapy. Therefore, ideally, a prediction model needs to be developed that can be used intraoperatively.

The one-step nucleic acid amplification (OSNA) assay is a rapid molecular procedure for the detection of LN metastasis targeting cytokeratin 19 (CK19) messenger RNA (mRNA) that can be completed intraoperatively within 30–40 min.^19^,^20^ The diagnostic accuracy of OSNA has repeatedly been shown to be equivalent to that of routine histological examinations, including immunohistochemistry, using formalin-fixed paraffin-embedded (FFPE) lymph nodes (LNs), and is better than that of intraoperative frozen section examination.^21^–^23^ Construction of an intraoperative prediction model using OSNA is thus more promising than using frozen section examination. Rubio et al. introduced a nomogram using OSNA for predicting NSLN metastasis.^24^ However, since their nomogram includes pathological parameters that are only obtainable postoperatively, it cannot be used intraoperatively. In this study, we therefore aimed to develop an intraoperative prediction model for NSLN metastasis and four or more ALN metastases that uses OSNA and parameters obtainable both preoperatively and intraoperatively.

## Patients and Methods

This multicentric, retrospective study was conducted using data registered in ‘LYNOLOG’ (run by the Japanese Association for Theranostics), a database for breast cancer patients who underwent OSNA that also includes their clinicopathological data. This study included 4367 consecutive patients registered in ‘LYNOLOG’ from seven participating Japanese hospitals between May 2009 and February 2016.

We identified 623 of these patients as having primary invasive carcinoma (T1-3, N0, M0) based on the following criteria: successful SLNB with at least one positive SLN, and cALND with removal of at least five nodes. Patients who had undergone neoadjuvant chemotherapy or hormonal therapy, as well as those with cALND with less than five lymph nodes removed, were excluded. Patients with an OSNA evaluation of + i were also excluded from the present analysis since an accurate CK19 mRNA copy number could not be determined. This study was approved by the Institutional Review Board of each participating hospital.

### Sentinel Lymph Node (SLN) Biopsy

All patients underwent SLNB with a combination of blue dye and/or radiotracer using different methods, including type of tracer and injection site, depending on the participating hospitals.^3^–^5^ cALND was performed if SLN was positive for macrometastasis, micrometastasis, or + i determined by OSNA.

### One-Step Nucleic Acid Amplification

Whole SLNs were subjected to intraoperative OSNA at six institutions, with the exception of one hospital where a 1-mm-thick slice was removed from the middle of each SLN for histological examination prior to OSNA. OSNA for the detection of SLN metastasis was performed as previously described in detail.^19^ The results were reported, according to the manufacturer’s instructions (Sysmex, Kobe, Japan), as negative (< 2.5 × 10^2^ copies/µL), + positive (≥ 2.5 × 10^2^ and < 5.0 × 10^3^ copies/µL = micrometastasis), ++positive (≥ 5.0 × 10^3^ copies/µL = macrometastasis), or + i (inhibited in the regular sample and > 2.5 × 10^2^ copies/µL in the diluted sample). As previously described, total tumor load (TTL) was defined as the total of CK19 mRNA copy numbers of each positive SLN.^25^

### Statistical Analysis

R software (version 3.4.0.) was used for all statistical analyses. The Chi square test and Fisher’s exact test were used for comparing the clinicopathological factors for both the training and validation groups. Differences in the mean values of continuous variables were analyzed using Welch’s *t* test. For the training group, logistic regression was used to analyze the association of each variable with the likelihood of NSLN metastasis or four or more ALN metastases. For the multivariate analysis, a backwards elimination procedure was used to drop variables in Wald tests (*p* value > 0.1). A nomogram for predicting NSLN metastasis or four or more ALN metastases was constructed with the remaining variables by means of the Wald test results for patients in the training group, and then validated using patients in the validation group. The nomogram was evaluated using the area under the receiver operating characteristic (ROC) curve (AUC), and DeLong’s test was used for comparison of ROC curves for the two models. A *p* value < 0.05 was considered significant.

## Results

### Construction of an Intraoperative Prediction Model (Nomogram) for Non-SLN (NSLN) Metastasis in Patients with Positive Sentinal Lymph Nodes (SLNs)

**Fig. 1:**
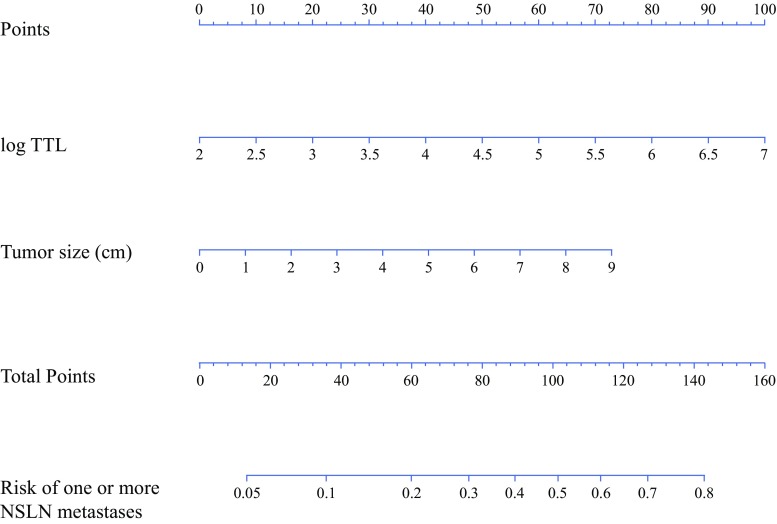
Nomogram for prediction of NSLN metastasis. *NSLN* non-sentinel lymph node, *TTL* total tumor load

The 623 patients were randomly divided into two cohorts—the training cohort (*n* = 312) and the validation cohort (*n* = 311). The clinicopathological parameters of the patients in these two cohorts were well balanced (Table 1), with 490 patients (78.7%) having one positive SLN, 103 (16.5%) having two positive SLNs, and 30 (4.8%) having three or more positive SLNs. Univariate analysis (logistic regression analysis) of the clinicopathological parameters available before and during surgery revealed that for the training cohort, clinical tumor size, histological grade, number of positive SLNs, number of macrometastatic SLNs, and TTL were significantly associated with NSLN metastasis (electronic supplementary Table 1). Multivariate analysis (a backward elimination procedure with Wald tests) of these five parameters showed that TTL and clinical tumor size were significantly associated with NSLN metastasis. These two factors were then used to construct the intraoperative nomogram for the prediction of NSLN metastasis (Fig. 1) The AUCs of the ROC analysis of this nomogram were 0.71 and 0.70 for the training and validation cohorts, respectively (electronic supplementary Fig. 1). The cut-off for the training cohort value was determined so that the negative predictive value (NPV) would be more than 90%, resulting in an NPV of 93.2% (95% confidence interval [CI] 87.4–98.9%) using the cut-off value of 10% for the validation cohort (electronic supplementary Table 2).

**Table 1:** Patient characteristics

Characteristic	Total [*n* = 623]	Training [*n* = 312]	Validation [*n* = 311]	*p* value
Age, years [median (range)]	53 (28–90)	53 (28–86)	54 (29–90)	0.954^a^
Menopausal status				
Premenopausal	283 (45.4)	145 (46.5)	138 (44.4)	0.655^b^
Postmenopausal	340 (54.6)	167 (53.5)	173 (55.6)	
Surgery				
Partial mastectomy	347 (55.7)	172 (55.1)	175 (56.3)	0.837^b^
Total mastectomy	276 (44.3)	140 (44.9)	136 (43.7)	
SLNB method				
RI and dye	500 (80.3)	244 (78.2)	256 (82.3)	0.428^b^
RI	32 (5.1)	17 (5.4)	15 (4.8)	
Dye	78 (12.5)	44 (14.1)	34 (10.9)	
Unknown	13 (2.1)	7 (2.2)	6 (1.9)	
Clinical tumor size, cm				
Mean ± SD	2.1 ± 1.2	2.1 ± 1.1	2.1 ± 1.3	0.533^a^
Unknown	7 (1.1)	3 (1.0)	4 (1.3)	
cT				
T1	366 (58.7)	183 (58.7)	183 (58.8)	0.294^b^
T2	241 (38.7)	124 (39.7)	117 (37.6)	
T3	16 (2.6)	5 (1.6)	11 (3.5)	
ER				
Positive	551 (88.4)	274 (87.8)	277 (89.1)	0.774^c^
1–9%	8 (1.3)	5 (1.6)	3 (1.0)	
Negative	64 (10.3	33 (10.6)	31 (10.0)	
PR				
Positive	457 (73.4)	231 (74.0)	226 (72.7)	0.467^b^
1–9%	35 (5.6)	14 (4.5)	21 (6.8)	
Negative	131 (21.0)	67 (21.5)	64 (20.6)	
HER2				
Positive	79 (12.7)	37 (11.9)	42 (13.5)	0.619^b^
Negative	536 (86.0)	271 (86.9)	265 (85.2)	
Unknown	8 (1.3)	4 (1.3)	4 (1.3)	
Tumor type				
Invasive ductal	568 (91.2)	284 (91.0)	284 (91.3)	0.982^b^
Invasive lobular	27 (4.3)	14 (4.5)	13 (4.2)	
Special type	28 (4.5)	14 (4.5)	14 (4.5)	
Histological grade				
1	240 (38.5)	126 (40.4)	114 (36.7)	0.428^b^
2	272 (43.7)	128 (41.0)	144 (46.3)	
3	102 (16.4)	53 (17.0)	49 (15.8)	
Unknown	9 (1.4)	5 (1.6)	4 (1.3)	
No. of SLNs				
1	234 (37.6)	115 (36.9)	119 (38.3)	0.599^b^
2	210 (33.7)	111 (35.6)	99 (31.8)	
≥ 3	179 (28.7)	86 (27.6)	93 (29.9)	
No. of positive SLNs				
1	490 (78.7)	245 (78.5)	245 (78.8)	0.232^b^
2	103 (16.5)	56 (17.9)	47 (15.1)	
≥3	30 (4.8)	11 (3.5)	19 (6.1)	
No. of NSLNs				
5–9	179 (28.7)	90 (28.8)	89 (28.6)	0.997^b^
10–14	216 (34.7)	107 (34.3)	109 (35.0)	
15–19	130 (20.9)	66 (21.2)	64 (20.6)	
≥ 20	98 (15.7)	49 (15.7)	49 (15.8)	
No. of positive NSLNs				
0	484 (77.7)	242 (77.6)	242 (77.8)	0.363^c^
1	57 (9.1)	23 (7.4)	34 (10.9)	
2–3	45 (7.2)	26 (8.3)	19 (6.1)	
4–9	28 (4.5)	15 (4.8)	13 (4.2)	
≥ 10	9 (1.4)	6 (1.9)	3 (1.0)	
Log TTL				
Mean ± SD	4.1 ± 1.1	4.2 ± 1.0	4.1 ± 1.1	0.153^a^

Data are expressed as *n* (%) unless otherwise specified

*SLNB* sentinel lymph node biopsy, *RI* radioisotope, *cT* clinical T stage, *ER* estrogen receptor, *PR* progesterone receptor, *HER2* human epidermal growth factor receptor 2, *SLNs* sentinel lymph nodes, *NSLN* non-sentinel lymph nodes, *TTL* total tumor load

^a^Welch’s *t* test

^b^Chi square test

^c^Fisher’s exact test

### Comparison of our Nomogram with Other Models for the Prediction of NSLN Metastasis

**Fig. 2:**
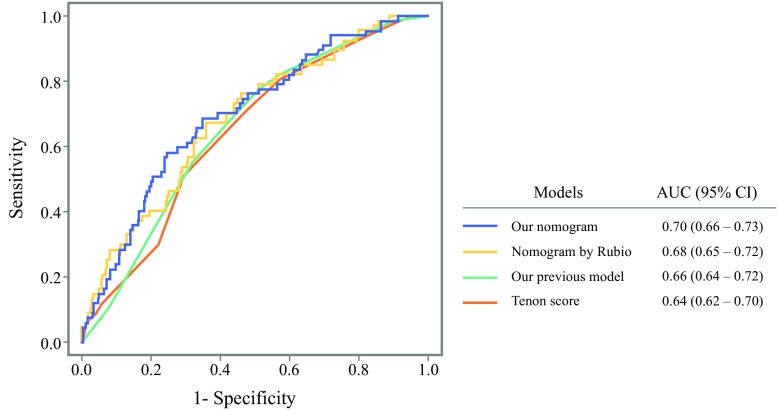
Comparison of receiver operating characteristic curves of prediction models for NSLN metastasis. *AUC* area under the curve, *CI* confidence interval

The validation cohort was used for comparing our nomogram for the prediction of NSLN metastasis with other prediction models. As shown in Fig. 2, the AUC of the ROC curve of our nomogram (AUC 0.70) was as accurate as that of our previous model (AUC 0.66, *p* = 0.10)^26^ and the nomogram reported by Rubio et al. (AUC 0.68, *p* = 0.56),^24^ but marginally significantly better than that of the Tenon score (AUC 0.64, *p* = 0.054).^10^

### Construction of an Intraoperative Prediction Model (Nomogram) for Four or More Axillary Lymph Node (ALN) Metastases in Patients with One to Three Positive SLNs

**Fig. 3:**
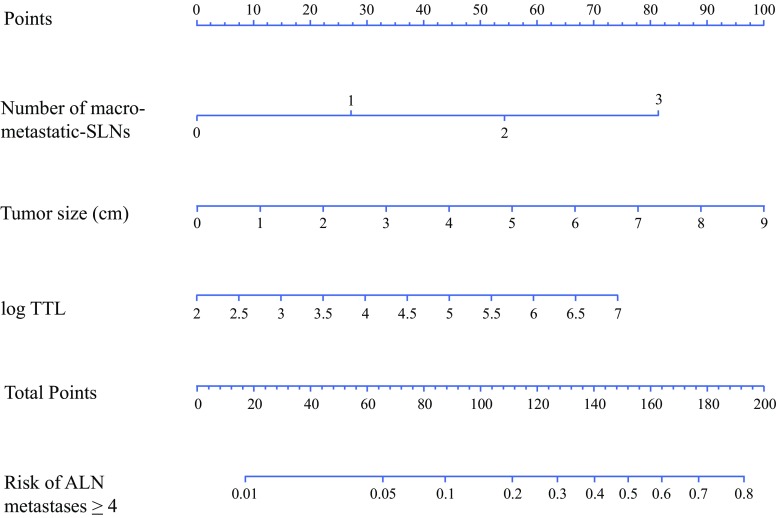
Nomogram for prediction of four or more ALN metastases. *SLNs* sentinel lymph nodes, *ALN* axillary lymph node, *TTL* total tumor load

Univariate analysis of the clinicopathological parameters available before and during surgery revealed that clinical tumor size, histological grade, number of positive SNs, number of macrometastatic SLNs, and TTL were significantly associated with four or more ALN metastases in the training cohort. Multivariate analysis of these five parameters demonstrated that clinical tumor size, number of macrometastatic SLNs, and TTL were significantly associated with four or more ALN metastases in the training cohort (electronic supplementary Table 3). These three factors were used to construct the intraoperative nomogram for the prediction of four or more ALN metastases (Fig. 3). The AUCs of ROC analysis of this nomogram were 0.79 and 0.70 for the training and validation cohorts, respectively (electronic supplementary Fig. 2). The cut-off value for the training cohort was determined so that the NPV would be more than 90%, resulting in an NPV of 96.9% (95% CI 94.4–99.3) using the cut-off value of 10% for the validation cohort (electronic supplementary Table 4).

### Comparison of our Nomogram with Other Models for the Prediction of Four or More ALN Metastases

**Fig. 4:**
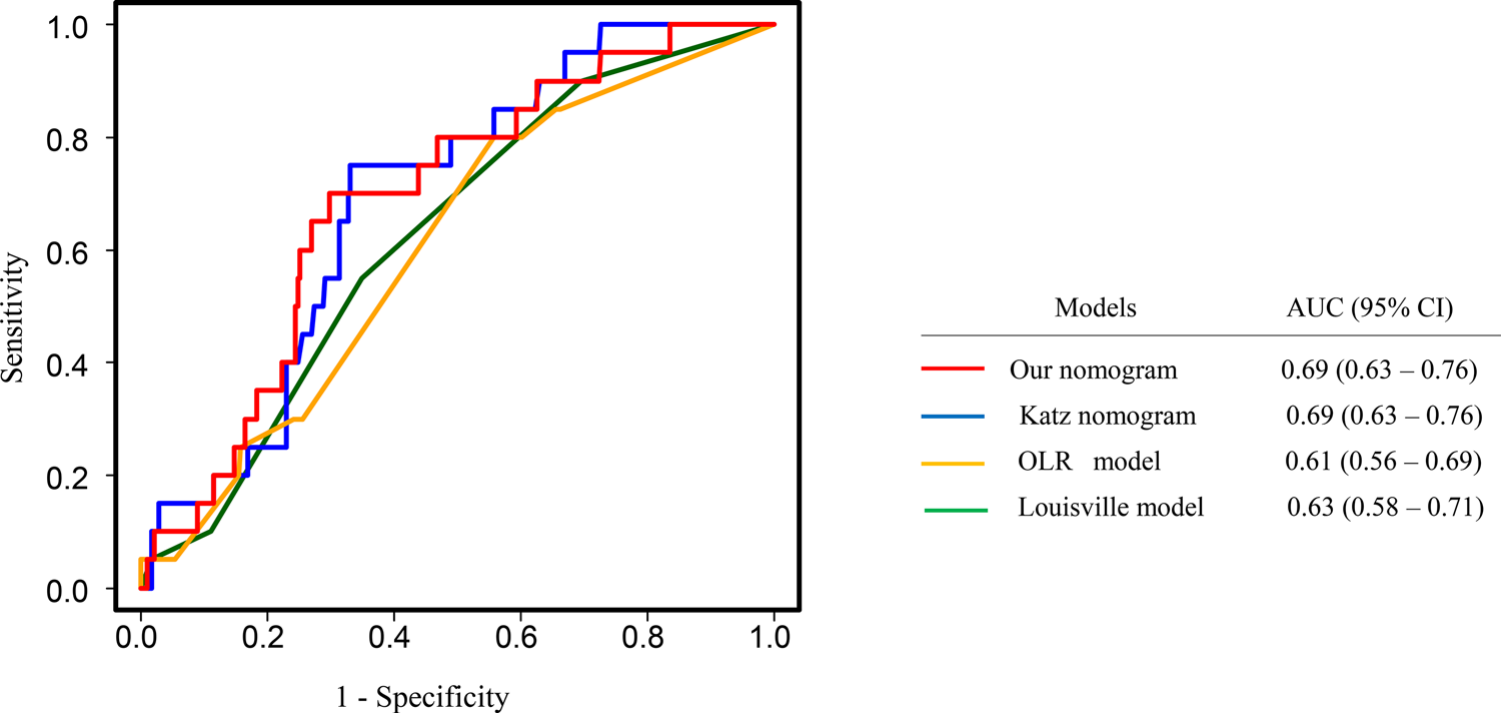
Comparison of receiver operating characteristic curves of prediction models for ALN metastasis ≥ 4. *ALN* axillary lymph node, *AUC* area under the curve, *CI* confidence interval

The validation cohort was used for comparing our nomogram for prediction of four or more ALN metastases with other prediction models. As shown in Fig. 4, the AUC of the ROC curve of our nomogram (AUC 0.69) was as accurate as that of the Katz nomogram (AUC 0.69, *p* = 0.99)^16^ and the Louisville score (excluding detection method) by Chagpar et al. (AUC 0.63, *p* = 0.29),^17^ and significantly better than the optimal logistic regression (OLR) model by Werkoff et al. (AUC 0.61, *p* = 0.002).^18^

## Discussion

Although intraoperative assessment of SLNs allows for immediate cALND in case of positive SLNs, and can thus avoid the second surgery, the sensitivity of the frozen section examination or imprint cytology varies from only 44–75%. Therefore, postoperative assessment using the FFPE LNs is preferable to the intraoperative pathological examination, although a significant proportion of patients who are assessed postoperatively as having SLN metastasis need to undergo the second surgery for cALND. However, OSNA renewed our interest in intraoperative assessment of SLNs, since OSNA has been repeatedly shown to be at least as accurate as histological examination, including immunohistochemistry, using the FFPE LNs,^21^,^22^ and better than frozen section examination or imprint cytology.^21^,^23^ These results point to the possibility that an intraoperative prediction model for NSLN metastasis as good as the postoperative prediction model can be constructed if OSNA is incorporated in the model.

Peg et al. first reported the concept of TTL, defined as the amount of CK19 mRNA copies in all positive SLNs,^25^ and concluded that TTL was an independent predictor of NSLN metastasis by using multivariate analysis of tumor size, human epidermal growth factor receptor 2 (HER2), LVI, and the number of metastatic SLNs. The result was consistent with that reported by Nabais et al.^27^ TTL comprises two elements (the number and the size of SLN metastases), indicating that TTL can accurately estimate axillary tumor burden as one continuous variable. Moreover, the advantage of TTL as a predictive parameter is that TTL is automatized, reproducible, and can be assessed intraoperatively. Many investigators have suggested that the size of metastasis in SLN could be an important factor in predicting NSLN metastasis.^14^ However, it has also been suggested that diagnostic reproducibility for pathologic classification of small metastases, unlike that of OSNA, has proven to be poor in routine practice,^28^ thus making the incorporation of OSNA into the nomogram rational.

According to the AUC of the ROC curve, the predictive accuracy of our nomogram for NSLN metastasis was 0.70, and 23.8% of patients could be classified into the low-risk group for NSLN metastasis with a high NPV of 93.2% (electronic supplementary Table 2). Although the population of the low-risk group is small, it is thought that cALND could be avoided more for patients in the low-risk group than those in the high-risk group (probability of NSLN metastasis: 6.8 vs. 27.4%, *p* = 0.0002). We compared the results for our nomogram with those of previously reported nomograms (Fig. 2), and found our nomogram to be as good as the others; however, only our nomogram could be used intraoperatively, and thus has the advantage of avoiding a second surgery for a significant proportion of patients.

The ACOSOG Z0011 trial has demonstrated that cALND can be avoided for selected SLN-positive patients.^6^, ^7^ However, 56% of patients included in this study did not meet the Z0011 criteria. Similarly, Berrang et al. reported that nearly 60% of SLN-positive patients did not meet the Z0011 criteria.^29^ Furthermore, it has been claimed that the application of Z0011 could lead to the avoidance of cALND in only < 10% of all SLNB cases.^30^ Thus, for a majority of SLN-positive patients, cALND is still the standard procedure. These results seem to highlight the importance of an effective nomogram for NSLN metastasis, even in the post-Z0011 era.

Nonetheless, it is expected that cALND can be more and more frequently avoided in the future for SLN-positive breast cancer patients with favorable tumor characteristics such as small size, ER-positivity, and low grade, even if their NSLN metastasis status is unknown. However, we believe that it is important for such patients to estimate their total number of ALN metastases and, more exactly, to estimate whether the total number is less than or equal to, or more than four since the regimens of adjuvant chemotherapy, as well as indications for radiation therapy to the regional LNs, can be affected by this estimation.^31^ In fact, some studies have described predictive, although postoperative, models for four or more ALNs.^16^–^18^ We have therefore developed an intraoperative nomogram for four or more ALN metastases, which proved to be capable of classifying 63.5% of patients into the low-risk group with only a 3.1% chance of having metastasis ≥ 4 (electronic supplementary Table 4). The AUC of our nomogram (0.70) was similar to that of the previously reported postoperative models (Fig. 4). For patients with favorable tumor characteristics, the total number of ALN metastases seems to be very important for decision making regarding regimens of adjuvant chemotherapy and indication of radiation therapy for regional LNs.

Since it has been reported that the frequency of lymph node metastasis differs depending on subtype, ideally it is necessary to verify the usefulness of our developed nomograms for each subtype. However, unfortunately, because the number of patients with HER2-positive or triple-negative breast cancer is too small, meaningful analysis cannot be carried out in these subtypes (electronic supplementrary Figs. 3, 4 and 5). Regarding ER-positive/HER2-negative subtype, the AUC in the prediction of NSLN metastasis was 0.68, and the AUC in the prediction of four or more ALN metastases was 0.69 (electronic supplementary Figs. 6 and 7).

In the development of a nomogram for the prediction of NSLN metastasis, the number of NSLNs obtained at cALND is important since the NSLN status is unlikely to be assessed correctly if the number of NSLNs is too small. In the present study, we included patients with at least five NSLNs. This inclusion criteria was the same as that reported by Rubio et al., who first developed a nomogram based on TTL by OSNA.^24^ In addition, the other studies also adopted a similar criteria in that patients with at least three to four NSLNs could be included^11^–^13^ (electronic supplementary Table 5). Furthermore, the median number of NSLNs in our study was 13, similar to that reported by other studies, ranging from 5 to 40 (electronic supplementary Table 5). Therefore, we believe that the inclusion criteria adopted in our study are reasonable.

## Conclusions

We have succeeded in constructing intraoperative prediction nomograms for NSLN metastasis and four or more ALN metastases, and have demonstrated that both are as accurate as the predictions obtained from previously reported postoperative models. These two nomograms are thought to be useful not only for patients who do not meet the Z0011 criteria but also for those who meet the criteria and for whom ALND can be avoided, because they provide information as to the total number of ALN metastases and can help reach decisions regarding adjuvant therapies. Nevertheless, our findings presented here need to be validated in future studies using larger numbers of patients.

## Electronic supplementary material

Below is the link to the electronic supplementary material.

Supplementary material 1 (DOCX 50 kb)

Supplementary material 2 (DOCX 930 kb)
