# Supplementary material for: Intraoperative Nomograms, Based on One-Step Nucleic Acid Amplification, for Prediction of Non-sentinel Node Metastasis and Four or More Axillary Node Metastases in Breast Cancer Patients with Sentinel Node Metastasis
Source: Ann Surg Oncol. 2018 Jul 5;25(9):2603–11. doi: 10.1245/s10434-018-6633-0 (PMC6097722; doi:10.1245/s10434-018-6633-0)
Supplement: Supplementary file 1 — Supplementary material 1 (DOCX 50 kb) [file 10434_2018_6633_MOESM1_ESM.docx]

| Supplementary Table 1. Logistic regression analysis of parameters associated with non-SLN metastasis for patients with positive SLN for the training cohort. | | | | |
| --- | --- | --- | --- | --- |
|  |  | **Non-SLN metastasis** | |  |
| **Characteristic** | **Total** | **Negative** | **Positive** | **P value** |
|  | **(*n* = 312)** | **(*n* = 242)** | **(*n* = 70)** |  |
| **Age** | | | | |
| Median | 53.0 (28.0 - 86.0) | 53.0 (31.0 - 86.0) | 51.0 (28.0 - 81.0) | 0.464 |
| **Menopausal status** | | | | |
| Premenopausal | 145 (46.5 %) | 112 (46.3 %) | 33 (47.1 %) | 0.899 |
| Postmenopausal | 167 (53.5 %) | 130 (53.7 %) | 37 (52.9 %) |  |
| **Clinical tumor size (cm)** | | | | |
| Mean | 2.1 ± 1.1 | 2.0 ± 1.1 | 2.3 ± 1.2 | 0.025 |
| **cT** | | | | |
| T1 | 183 (58.7 %) | 148 (61.2 %) | 35 (50.0 %) | 0.089 |
| T2 | 124 (39.7 %) | 90 (37.2 %) | 34 (48.6 %) |  |
| T3 | 5 (1.6 %) | 4 (1.7 %) | 1 (1.4 %) |  |
| **ER** | | | | |
| Positive | 279 (89.4 %) | 217 (89.7 %) | 62 (88.6 %) | 0.793 |
| Negative | 33 (10.6%) | 25 (10.3%) | 8 (11.4%) |  |
| **PR** | | | | |
| Positive | 245 (78.5 %) | 190 (78.5 %) | 55 (78.6 %) | 0.992 |
| Negative | 67 (21.5%) | 52 (21.5%) | 15 (21.4%) |  |
| **HER2** | | | | |
| Positive | 37 (12.0 %) | 28 (11.7 %) | 9 (13.0 %) | 0.765 |
| Negative | 271 (88.0%) | 211 (88.3%) | 60 (87.0%) |  |
| **Tumor type** | | | | |
| Invasive ductal | 284 (91.0 %) | 222 (91.7 %) | 62 (88.6 %) | 0.555 |
| Invasive lobular | 14 (4.5 %) | 10 (4.1 %) | 4 (5.7 %) |  |
| Special type | 14 (4.5 %) | 10 (4.1 %) | 4 (5.7 %) |  |
| **Histological Grade** | | | | |
| Grade1 | 126 (41.0 %) | 103 (43.1 %) | 23 (33.8 %) | 0.047 |
| Grade2 | 128 (41.7 %) | 91 (38.1 %) | 37 (54.4 %) |  |
| Grade3 | 53 (17.3 %) | 45 (18.8 %) | 8 (11.8 %) |  |
| **No. of positive SLNs** | | | | |
| 1 | 245 (78.5 %) | 196 (81.0 %) | 49 (70.0 %) | 0.043 |
| 2 | 56 (17.9 %) | 39 (16.1 %) | 17 (24.3 %) |  |
| 3 | 9 (2.9 %) | 6 (2.5 %) | 3 (4.3 %) |  |
| 5 | 1 (0.3 %) | 1 (0.4 %) | 0 (0 %) |  |
| 6 | 1 (0.3 %) | 0 (0 %) | 1 (1.4 %) |  |
| **No. of macro-metastatic SLNs** | | | | |
| 0 | 111 (35.6 %) | 100 (41.3 %) | 11 (15.7 %) | < 0.0001 |
| 1 | 162 (51.9 %) | 120 (49.6 %) | 42 (60.0 %) |  |
| 2 | 35 (11.2 %) | 21 (8.7 %) | 14 (20.0 %) |  |
| 3 | 3 (1.0 %) | 0 (0 %) | 3 (4.3 %) |  |
| 4 | 1 (0.3 %) | 1 (0.4 %) | 0 (0 %) |  |
| **log TTL** | | | | |
| Mean | 4.2 ± 1.0 | 4.0 ± 1.0 | 4.7 ± 1.0 | < 0.0001 |

SLN, Sentinel lymph node; cT, Clinical T stage; ER, Estrogen receptor; PR, Progesterone receptor, HER2, Human epidermal growth factor receptor; NSLN, non- Sentinel lymph node; TTL, Total tumor load

Supplementary Table 2. Results of prediction by the nomogram of one or more NSLN metastases in validation cohort

|  | Nomogram | |  |
| --- | --- | --- | --- |
|  | Positive | Negative |  |
| Non-SLN |  |  |  |
| Positive | 64 | 5 |  |
| Negative | 170 | 68 |  |
| Accuracy = 43.0 %; Sensitivity = 92.8%; Specificity = 28.6 %, | | | |
| PPV = 27.4 %, NPV = 93.2 % [95 % CI: 87.4 - 98.9] | |  |  |
| *Positive: having a probability of NSLN metastasis of 10% or more predicted by the nomogram | | | |
| **Negative: having a probability of NSLN metastasis less than 10% predicted by the nomogram | | | |

| Supplementary Table 3. Logistic regression analysis of parameters associated with four or more axillary node metastases for patients with positive SLN for the training cohort. | | | | |
| --- | --- | --- | --- | --- |
|  |  | **4 or more ALNM** | |  |
| **Characteristic** | **Total** | **negative** | **positive** | **P value** |
|  | **(*n* = 310)** | **(*n* = 276)** | **(*n* = 34)** |  |
| **Age** | | | | |
| Median | 53.0 (28.0 - 86.0) | 53.0 (28.0 - 86.0) | 54.0 (30.0 - 78.0) | 0.642 |
| **Menopausal status** | | | | |
| Premenopausal | 143 (46.1 %) | 128 (46.4 %) | 15 (44.1 %) | 0.803 |
| Postmenopausal | 167 (53.9 %) | 148 (53.6 %) | 19 (55.9 %) |  |
| **Clinical tumor size (cm)** | | | | |
| Mean | 2.0 ± 1.1 | 2.0 ± 1.0 | 2.6 ± 1.5 | 0.008 |
| **cT** | | | | |
| T1 | 183 (59.0 %) | 168 (60.9 %) | 15 (44.1 %) | 0.075 |
| T2 | 122 (39.4 %) | 104 (37.7 %) | 18 (52.9 %) |  |
| T3 | 5 (1.6 %) | 4 (1.4 %) | 1 (2.9 %) |  |
| **ER** | | | | |
| Positive | 278 (89.7 %) | 248 (89.9 %) | 30 (88.2 %) | 0.77 |
| Negative | 32 (10.3%) | 28 (10.1%) | 4 (11.8%) |  |
| **PR** | | | | |
| Positive | 244 (78.7 %) | 216 (78.3 %) | 28 (82.4 %) | 0.583 |
| Negative | 66 (21.3%) | 60 (21.7%) | 6 (17.6%) |  |
| **HER2** | | | | |
| Positive | 37 (12.1 %) | 31 (11.4 %) | 6 (17.6 %) | 0.296 |
| Negative | 269 (87.9%) | 241 (88.6%) | 28 (82.4%) |  |
| **Tumor type** | | | | |
| Invasive ductal | 282 (91.0 %) | 253 (91.7 %) | 29 (85.3 %) | 0.635 |
| Invasive lobular | 14 (4.5 %) | 12 (4.3 %) | 2 (5.9 %) |  |
| Special type | 14 (4.5 %) | 11 (4.0 %) | 3 (8.8 %) |  |
| **Histological Grade** | | | | |
| Grade1 | 125 (41.0 %) | 117 (42.9 %) | 8 (25.0 %) | 0.033 |
| Grade2 | 127 (41.6 %) | 108 (39.6 %) | 19 (59.4 %) |  |
| Grade3 | 53 (17.4 %) | 48 (17.6 %) | 5 (15.6 %) |  |
| **No. of positive SLNs** | | | | |
| 1 | 245 (79.0 %) | 225 (81.5 %) | 20 (58.8 %) | 0.002 |
| 2 | 56 (18.1 %) | 45 (16.3 %) | 11 (32.4 %) |  |
| 3 | 9 (2.9 %) | 6 (2.2 %) | 3 (8.8 %) |  |
| **No. of macro-metastatic SLNs** | | | | |
| 0 | 111 (35.8 %) | 108 (39.1 %) | 3 (8.8 %) | < 0.0001 |
| 1 | 162 (52.3 %) | 144 (52.2 %) | 18 (52.9 %) |  |
| 2 | 35 (11.3 %) | 24 (8.7 %) | 11 (32.4 %) |  |
| 3 | 2 (0.6 %) | 0 (0 %) | 2 (5.9 %) |  |
| **log TTL** | | | | |
| Mean | 4.2 ± 1.0 | 4.1 ± 1.0 | 5.0 ± 0.8 | < 0.0001 |

SLN, Sentinel lymph node; ALNM, Axillary lymph node metastasis; cT, Clinical T stage; ER, Estrogen receptor; PR, Progesterone receptor, HER2, Human epidermal growth factor receptor; NSLN, non- Sentinel lymph node; TTL, Total tumor load

Supplementary Table 4. Results of prediction of 4 or more ALN metastasis in validation cohort by the nomogram

|  | Nomogram | |
| --- | --- | --- |
|  | Positive | Negative |
| Non-SLN |  |  |
| Positive | 15 | 6 |
| Negative | 95 | 185 |
| Accuracy = 66.4 %, Sensitivity = 71.4%, Specificity = 66.1 %, | | |
| PPV = 13.6 %, NPV =96.9 %, [95 % CI: 94.4 – 99.3] | | |
| *Positive: having a probability of four or more ALN metastases of 10% or more predicted by the nomogram | | |
| **Negative: having a probability of four or more ALN metastases of less than 10% predicted by the nomogram | | |

Supplementary Table 5. Comparison of number of NSLNs among studies on nomograms for prediction of NSLN metastasis

|  | Number of NSLNs | |
| --- | --- | --- |
| Studies | Median | Range |
| MDACC^11^ | 15 | 3-37 |
| Cambridge^12^ | 14 | 4-32 |
| Stanford Online^13^ | 21 | 4-52 |
| Tenon^10^ | 9 ^a^ | 3-21 |
| Our study | 13 | 5-40 |

NSLN, non- Sentinel lymph node; MDACC, The university of Texas MD Anderson Cancer Center

^a^ Mean
