# Supplementary material for: Intraoperative Nomograms, Based on One-Step Nucleic Acid Amplification, for Prediction of Non-sentinel Node Metastasis and Four or More Axillary Node Metastases in Breast Cancer Patients with Sentinel Node Metastasis
Source: Ann Surg Oncol. 2018 Jul 5;25(9):2603–11. doi: 10.1245/s10434-018-6633-0 (PMC6097722; doi:10.1245/s10434-018-6633-0)
Supplement: Supplementary file 2 — Supplementary material 2 (DOCX 930 kb) [file 10434_2018_6633_MOESM2_ESM.docx]

**Supplementary Figure Legends**

Supplementary Figure 1.


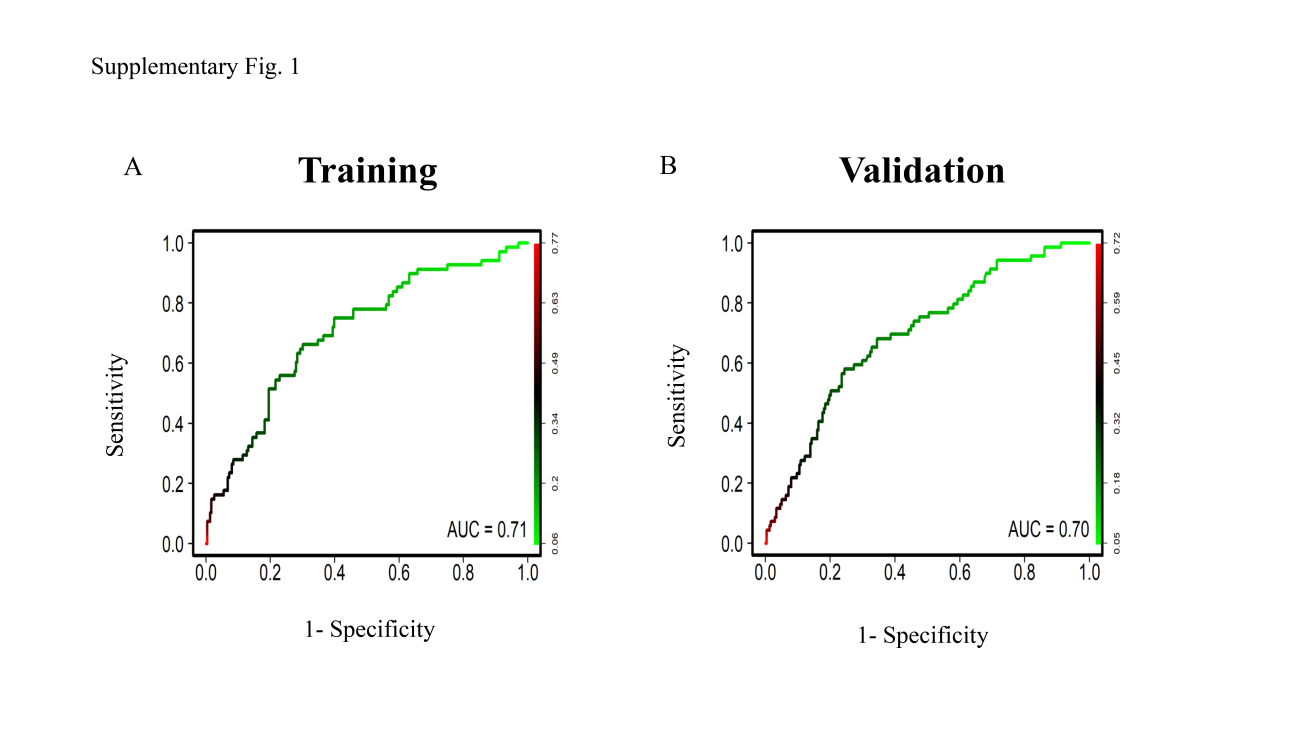


Receiver operating characteristics curve of our nomogram for prediction of one or more NSLN metastases for the training cohort (A) and the validation cohort (B). The colors of the graph correspond to the colors on the right vertical axis, which shows the value of the results calculated by the nomogram.

AUC, Area under the curve

Supplementary Figure 2.


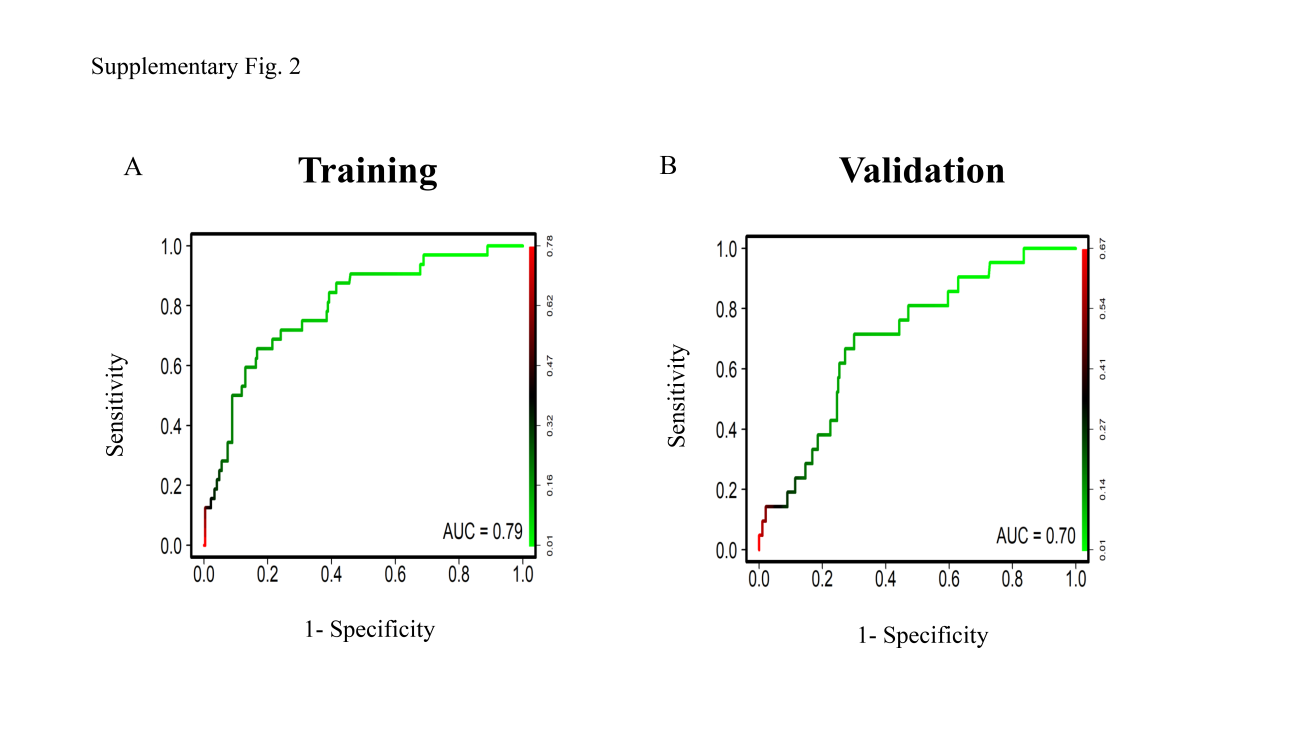


Receiver operating characteristics curve of our nomogram for prediction of four or more ALN metastases for the training cohort (A) and the validation cohort (B).

The colors of the graph correspond to the colors on the right vertical axis, which shows the value of the results calculated by the nomogram.

AUC, Area under the curve

Supplementary Figure 3.


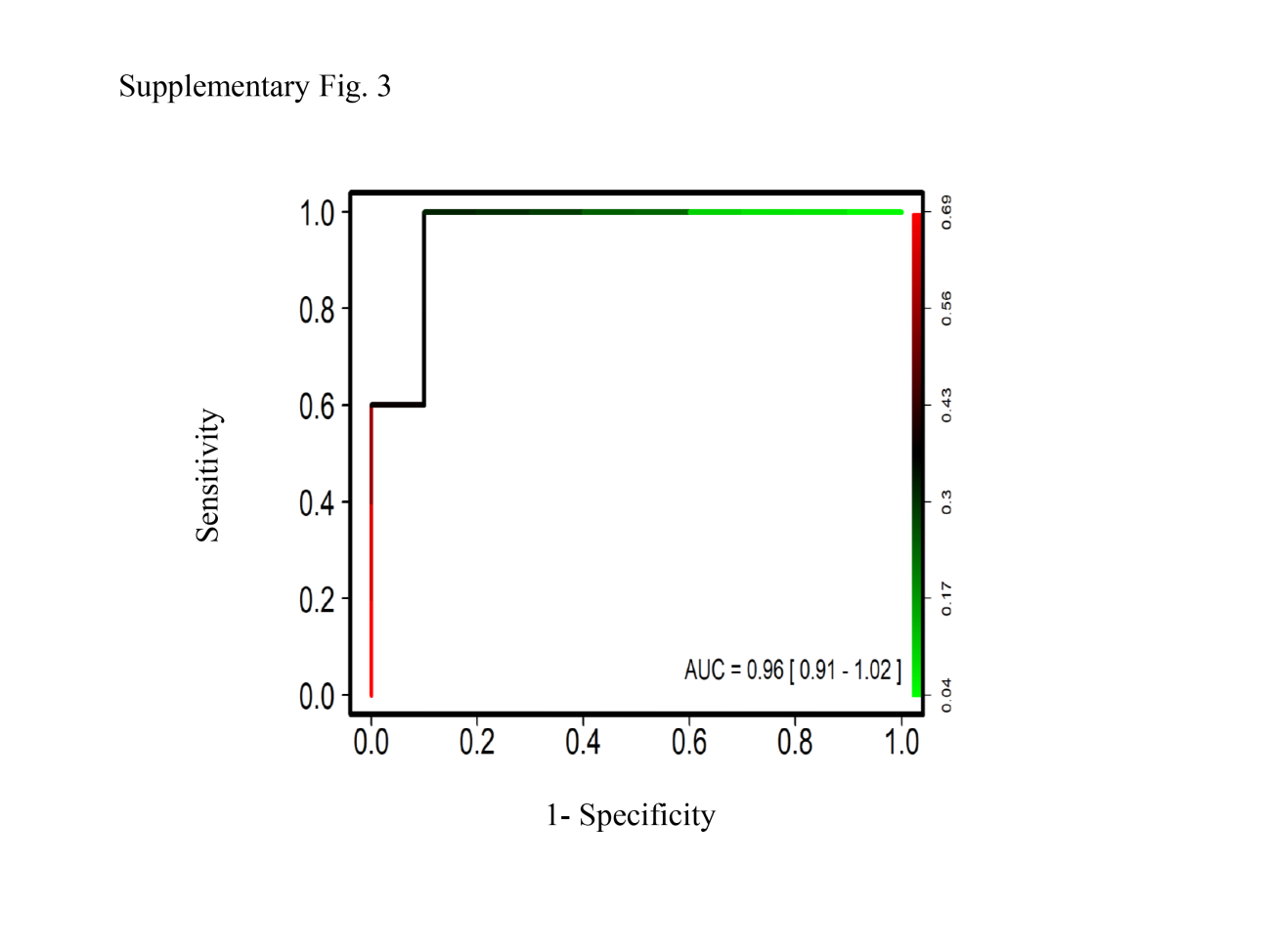


Receiver operating characteristics curve of our nomogram for prediction of one or more NSLN metastases for the validation cohort in patients with Her2-positive breast cancer.

The colors of the graph correspond to the colors on the right vertical axis, which shows the value of the results calculated by the nomogram.

AUC, Area under the curve

Supplementary Figure 4.


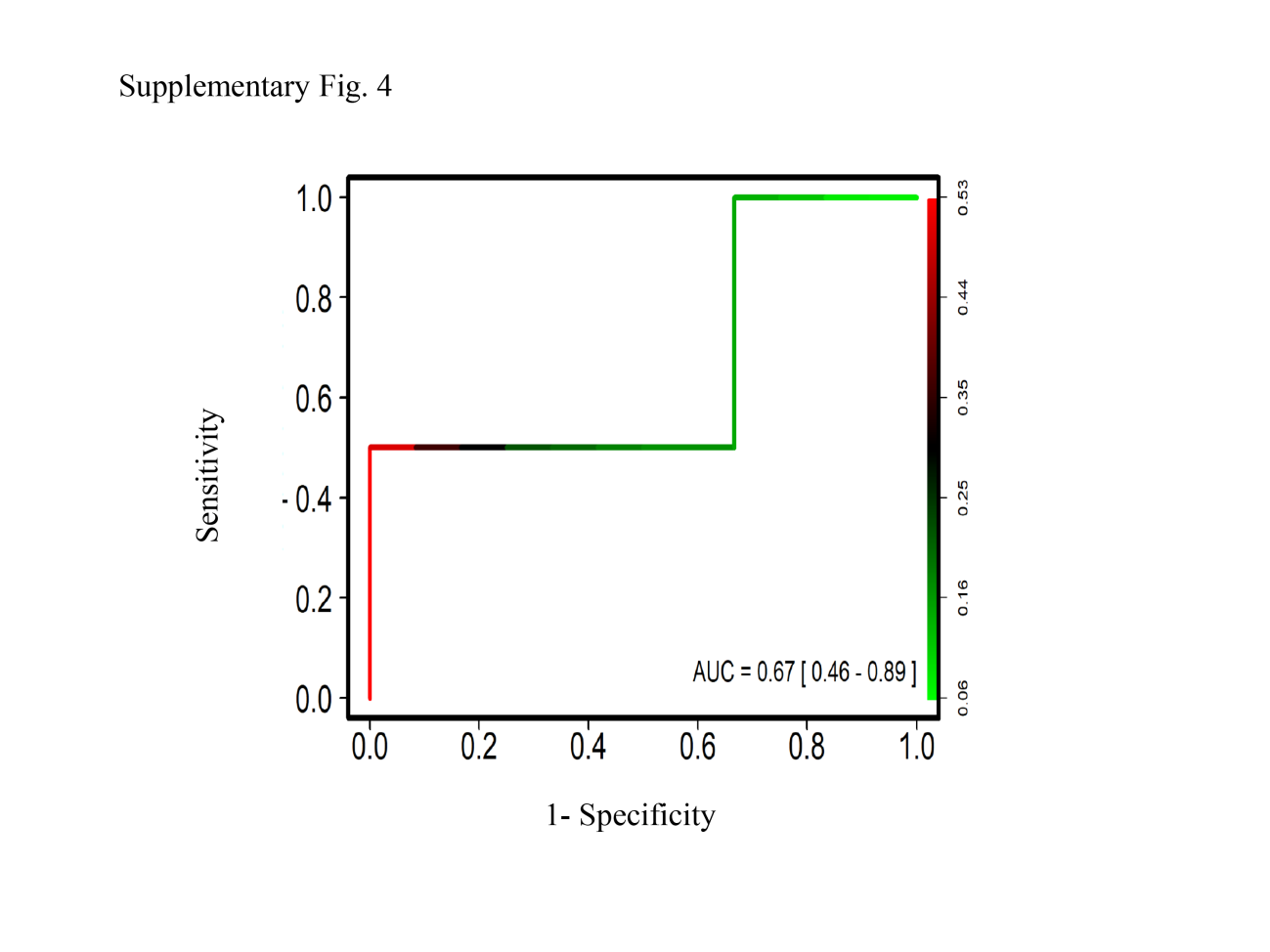


Receiver operating characteristics curve of our nomogram for prediction of one or more NSLN metastases for the validation cohort in patients with Triple Negative breast cancer.

The colors of the graph correspond to the colors on the right vertical axis, which shows the value of the results calculated by the nomogram.

AUC, Area under the curve

Supplementary Figure 5.


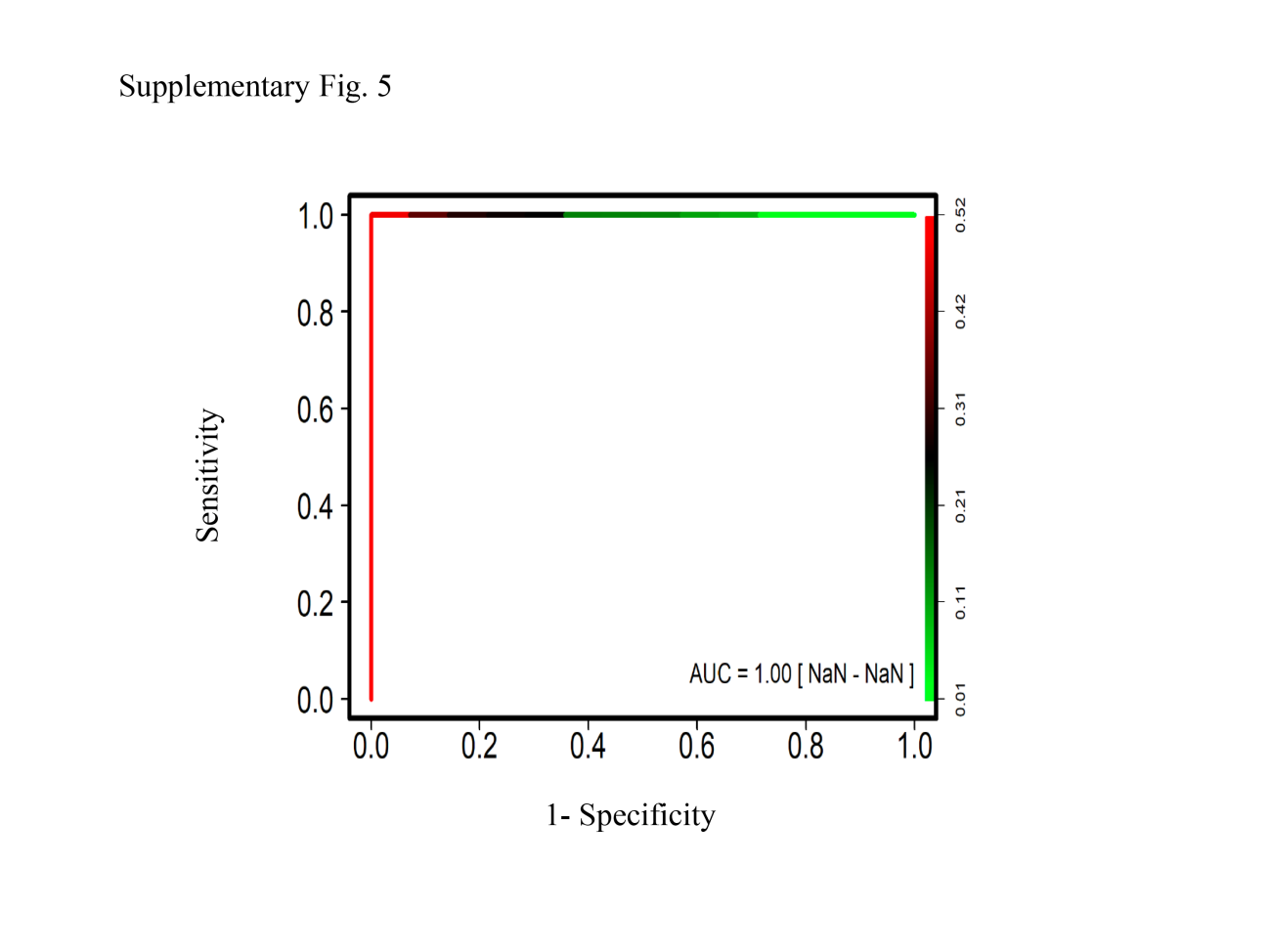


Receiver operating characteristics curve of our nomogram for prediction of four or more ALN metastases for the validation cohort in patients with Her2-positive breast cancer.

The colors of the graph correspond to the colors on the right vertical axis, which shows the value of the results calculated by the nomogram.

AUC, Area under the curve

Supplementary Figure 6.


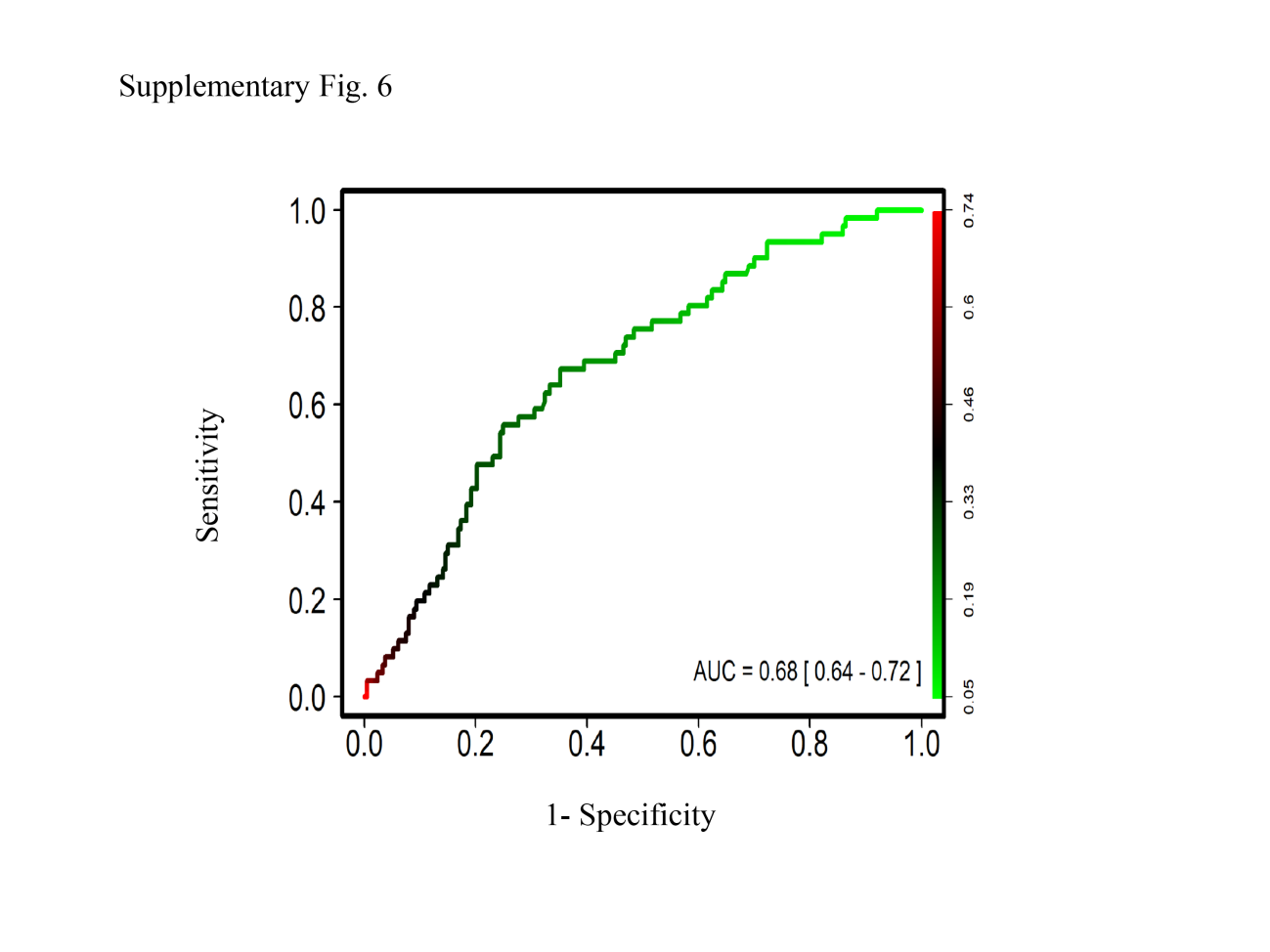


Receiver operating characteristics curve of our nomogram for prediction of one or more NSLN metastases for the validation cohort in patients with ER-positive/Her2-negative breast cancer.

The colors of the graph correspond to the colors on the right vertical axis, which shows the value of the results calculated by the nomogram.

AUC, Area under the curve

Supplementary Figure 7.


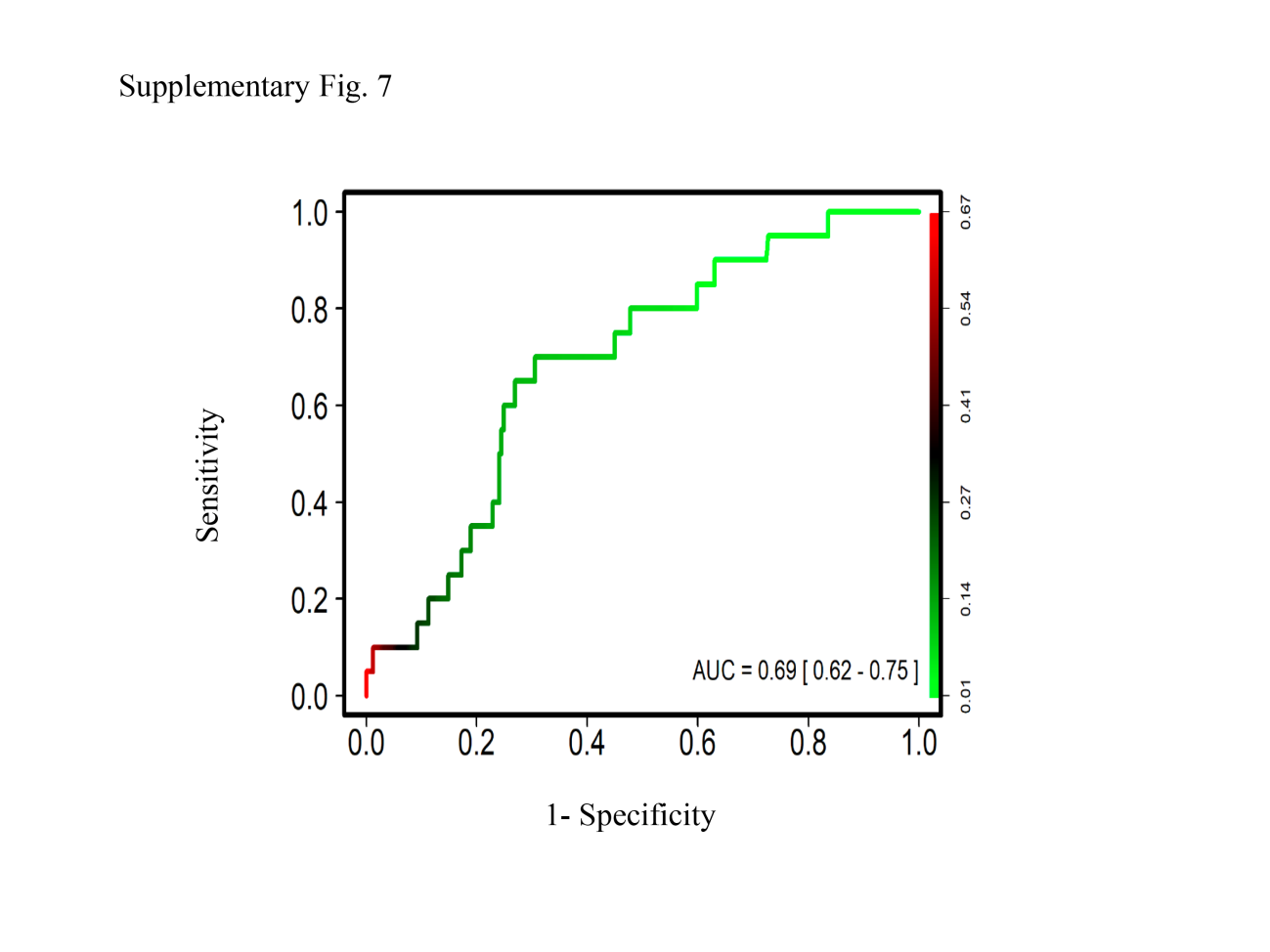


Receiver operating characteristics curve of our nomogram for prediction of four or more ALN metastases for the validation cohort in patients with ER-positive/Her2-negative breast cancer.

The colors of the graph correspond to the colors on the right vertical axis, which shows the value of the results calculated by the nomogram.

AUC, Area under the curve
